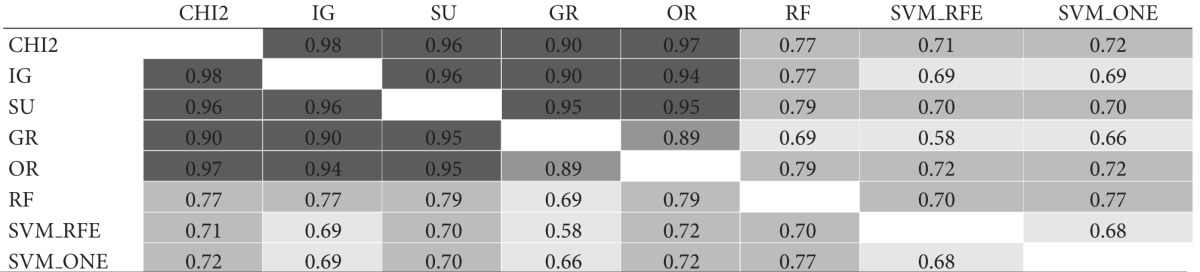# A Comparative Analysis of Biomarker Selection Techniques

**DOI:** 10.1155/2013/387673

**Published:** 2013-11-10

**Authors:** Nicoletta Dessì, Emanuele Pascariello, Barbara Pes

**Affiliations:** Dipartimento di Matematica e Informatica, Università degli Studi di Cagliari, Via Ospedale 72, 09124 Cagliari, Italy

## Abstract

Feature selection has become the essential step in biomarker discovery from high-dimensional genomics data. It is recognized that different feature selection techniques may result in different set of biomarkers, that is, different groups of genes highly correlated to a given pathological condition, but few direct comparisons exist which quantify these differences in a systematic way. In this paper, we propose a general methodology for comparing the outcomes of different selection techniques in the context of biomarker discovery. The comparison is carried out along two dimensions: (i) measuring the similarity/dissimilarity of selected gene sets; (ii) evaluating the implications of these differences in terms of both predictive performance and stability of selected gene sets. As a case study, we considered three benchmarks deriving from DNA microarray experiments and conducted a comparative analysis among eight selection methods, representatives of different classes of feature selection techniques. Our results show that the proposed approach can provide useful insight about the pattern of agreement of biomarker discovery techniques.

## 1. Introduction

Biomarker discovery from high-dimensional genomics data is a critical problem with numerous applications in biology and medicine, such as diagnosis and treatment of complex diseases at the molecular level. As reported in [1], a biomarker can be defined as “a characteristic that is objectively measured and evaluated as an indicator of normal biological processes, pathogenic processes, or pharmacological responses to a therapeutic intervention.” 

The discovery of biomarkers is typically modeled as a feature selection problem, where the aim is to identify the most discriminating features (i.e., genes) for a given classification task, for example, distinguishing between healthy and tumor tissues or between different tumor stages. While many feature selection techniques have been proposed [2], they do not necessarily identify the same feature subsets in the biomarker discovery process: indeed, even for the same data, different techniques can result in different groups of genes, raising questions about the biological significance of the discovered markers [3].

Surprisingly very few works in the literature have investigated, in a systematic way, the degree of similarity/dissimilarity between the outputs of different feature selection techniques in the context of biomarker discovery. Existing studies mostly focus on comparing the outcomes of different techniques in terms of predictive performance (see, e.g., [4, 5]), and, only recently, researchers have investigated the issue of stability of feature selection techniques with respect to sample variation [6, 7].

In this paper, we propose a general methodology for comparing different approaches to biomarker selection. The comparison is carried out along two dimensions: (i) measuring the similarity/dissimilarity of selected gene sets; (ii) evaluating the implications of these differences in terms of both predictive performance and stability of selected gene sets. As regards the similarity analysis, our methodology incorporates two ways of evaluating the degree of consistency among the gene sets: similarity in terms of *gene overlapping* and *functional similarity*. This twofold evaluation aims to investigate in what measure the biological functions captured by different gene sets can be similar, despite a limited overlapping among these sets. As regards the analysis of predictive performance and stability of selected biomarkers, our approach leverages on best practices from the literature [8, 9] and incorporates them into a unified comparative framework.

As a case study, we considered three benchmarks deriving from DNA microarray experiments, that is, the *Colon Tumor* dataset [10], the *Leukemia* dataset [11], and the *Prostate* dataset [12]. In the empirical analysis, eight selection methods were included as representative of different classes of feature selection techniques. Specifically, we considered both univariate approaches that evaluate the relevance of each single gene independently from the others and multivariate approaches that take into account interdependencies among genes. Our results show that the adopted methodology can provide useful insight about the pattern of agreement of different biomarker selection methods. 

The paper is organized as follows.  Section 2 details the methodology, motivating it in the context of the underlying background.  Section 3 illustrates the considered case study, describing the datasets, the selection methods, and the settings used in the experiments. The experimental results are presented and discussed in Section 4. Finally, Section 5 contains some final remarks and future research directions. 

## 2. Background and Methodology

In this study we focus on feature selection methods that produce a ranking of features based on their relevance for the predictive task at hand. Referred in the following as *rankers*, these methods assign a weight to each feature according to some scoring criterion that evaluates the degree of correlation between that feature and the target class. This weighting process can be carried out in two ways [2]: evaluating each feature independently from the others (univariate methods) or taking into account feature dependencies (multivariate methods). Once each feature has been weighted, a ranked list is produced where features appear in descending order of relevance: this list can be cut at a proper threshold point in order to obtain a subset of highly predictive features.

In the context of gene selection, the resulting feature subset can be interpreted as a signature that captures significant knowledge for a given diagnostic task. Our aim here is to compare, in a systematic way, the signatures produced by different rankers; this comparison is carried out along two dimensions, as detailed in the following.

### 2.1. Evaluating Similarity of Selected Gene Sets

**Figure 1:**
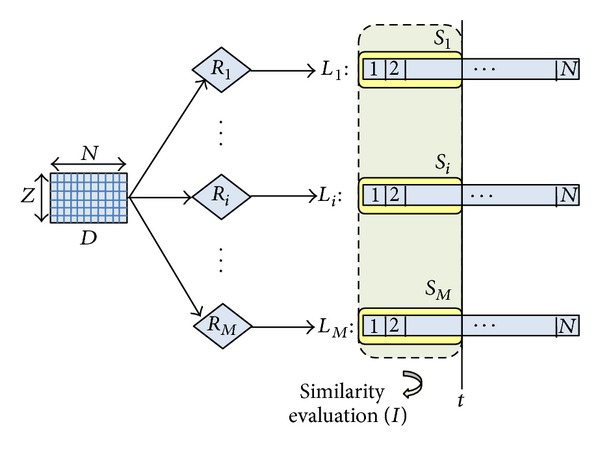
Similarity evaluation.

Given a dataset *D* with *Z* instances and *N* features (i.e., genes), a number *M* of rankers *R*_*i*_  (*i* = 1, 2, …, *M*) are applied to *D*: each *R*_*i*_ produces a ranked list *L*_*i*_  (*i* = 1, 2, …, *M*) where the *N* features appear in descending order of relevance. As illustrated in Figure 1, this results in *M* distinct ranked lists each expressing a different ordering of the *N* genes. 

When two lists *L*_*i*_ and *L*_*i*_  (*i*, *j* = 1, 2, …, *M*) are cut at a given threshold point *t*, the resulting gene sets *S*_*i*_ and *S*_*j*_  (*i*, *j* = 1, 2, …, *M*), of the same size *t*, can be compared according to some similarity index I. In particular, our methodology incorporates two approaches to measure the degree of similarity/dissimilarity among selected gene sets: similarity in terms of gene overlapping (I-overlap) and functional similarity(I-functional).

The similarity I-overlap between two sets *S*_*i*_ and *S*_*j*_ can be expressed as the number of genes that are present in both sets, that is, |*S*_*i*_∩*S*_*j*_|, properly normalized in the range [0,1], with 0 meaning that there is no overlap between the two sets and 1 that the two sets are identical. As normalization factor, we use |*S*_*i*_ ∪ *S*_*j*_|, as in [8]. After computing the I-overlap value for each pair of gene sets, we average over all pairwise similarity comparisons to obtain an overall evaluation of the degree of similarity between the *M* gene sets.

It has been observed, however, that the biological functions captured by different gene sets can be similar, despite a little degree of overlapping between these sets [13–15]. To compare two gene sets in functional terms, we exploit gene annotations from the Gene Ontology (GO) database [16], which provides a set of controlled vocabularies (biological or biochemical terms) describing gene products based on their functions in the cell. Specifically, for each gene set *S*_*i*_  (*i* = 1, 2,…, *M*), we extract the list of *molecular function* GO terms that annotate the *t* genes in the set. The resulting *M* lists of GO terms are then compared, in pairs, using the similarity measure (I-functional) proposed in [17] which considers not only the overlap between the lists but also the semantic relationships between GO terms.

### 2.2. Evaluating Predictive Performance and Stability of Selected Gene Sets

The predictive performance of a candidate gene set, that is, its capacity of discriminating a given target class (e.g., a pathological state), can be measured inducing a classification model on that set and using some test instances to evaluate this model in terms of metrics such as accuracy or ROC area [18]. This is usually done in a cross-validation setting, though it has been observed that it can produce overoptimistic results on small sample size domains [19].

Instead, no well-established evaluation protocol exists for measuring the stability of a biomarker selection algorithm, that is, its robustness with respect to sample variation: small changes in the original dataset should not affect the outcome of the selection process in a significant way. Research work on designing a suitable experimental procedure for testing stability in high-dimensional/small sample size domains is still ongoing [7], and in most cases stability is not evaluated in conjunction with predictive performance but in independent experiments.

**Figure 2:**
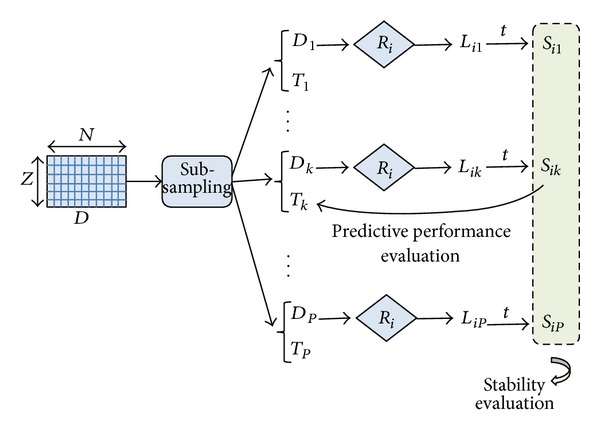
Joint evaluation of stability and predictive performance.

The methodology we adopt involves a single experimental setup to jointly evaluate both stability and predictive performance in the context of biomarker discovery. As illustrated in Figure 2, we extract from the original dataset *D*, with *Z* instances and *N* features (i.e., genes), a number *P* of reduced dataset *D*_*k*_  (*k* = 1, 2,…, *P*), each containing *f* · *Z* (with *f* ∈ (0,1)) instances randomly drawn from *D*.

Each of the previously considered rankers *R*_*i*_  (*i* = 1,2,…, *M*) is then applied to each reduced datasets *D*_*k*_  (*k* = 1,2,…, *P*) in order to obtain a ranked list *L*_*ik*_ and, after cutting the list at threshold *t*, a gene subset *S*_*ik*_. The *P* subsets selected by the ranker *R*_*i*_ from the *P* reduced datasets are then compared in terms of overlapping: the more similar (in average) these subsets are, the more stable the ranker *R*_*i*_ is.

We observe that the I-overlap measure is used in our approach in a twofold way: to compare the subsets produced by different rankers on the same dataset *D* and to compare the subsets produced by the same ranker on different reduced datasets drawn from *D*. Moreover, it should be observed that the recent literature suggests the Kuncheva index [20] as a more suitable similarity measure in the context of stability evaluation: it considers the degree of overlapping between two feature subsets and introduces a correction term that takes into account the probability that a feature is included in those subsets purely by chance. This correction term, however, does not affect the similarity value for feature subsets of small size, as the ones considered in the context of biomarker discovery [21].

To incorporate predictive performance evaluation in the above experimental protocol, we build on each reduced dataset *D*_*k*_  (*k* = 1,2,…, *P*) a classification model based on the gene set *S*_*ik*_ selected by the *i*th ranker: the model performance is then estimated on a test set *T*_*k*_ containing the fraction of instances of *D* not included in *D*_*k*_ (i.e., (1 − *f*) · *Z* instances). As performance metric we use the AUC (area under the ROC curve), as it synthesizes the information of sensitivity and specificity and provides a more reliable estimate in the case of unbalanced class distribution [22]. By averaging the AUC performance of the *P* classification models induced on the *P* gene subsets selected by the ranker *R*_*i*_, we can evaluate the effectiveness of that ranker in identifying highly predictive gene sets. This approach overcomes the risk of selection bias [23] since the test instances are not considered in any way in the gene selection stage. 

The above methodology ensures a joint evaluation of two fundamental requirements of any biomarker selection technique, that is, stability with respect to sample variation and effectiveness in terms of classification results, enabling the comparison of different techniques in a unified framework.

## 3. Case Study: Datasets and Settings

Consistently with the methodology described in Section 2, we conducted an empirical analysis on three benchmarks deriving from DNA microarray experiments.

- *Colon Tumor* dataset [10], containing 62 biological samples distinguished between tumor *colon* tissues (40 samples) and normal *colon* tissues (22 samples); each sample is described by the expression level of 2000 genes.
- *Leukemia* dataset [11], containing 72 samples belonging to patients suffering from acute myeloid *leukemia* (25 samples) and acute lymphoblastic *leukemia* (47 samples); each sample is described by the expression level of 7129 genes.
- *Prostate* dataset [12], containing 102 samples differed between healthy and tumor *prostate* tissues (50 and 52 samples, resp.); each sample is described by the expression level of 12600 genes.

The task, in terms of feature selection, is to identify the genes most useful in discriminating between cancerous and normal tissues (*Colon* and *Prostate* datasets) or between different tumor types (*Leukemia* dataset).

In our experiments, we compared *M* = 8 rankers that are representative of different classes of selection methods. In particular, we considered both univariate approaches, where each feature is ranked individually, and multivariate approaches that take into account feature dependencies.

Among the univariate techniques, we chose: chi Squared (*χ* ^2^) [24] as representative of statistic methods; information gain (IG) [25], symmetrical uncertainty (SU) [26], and gain ratio (GR) [27] as representatives of entropic methods; and finally OneR (OR) [28] as representative of methods that incorporate a classification technique (in this case, a simple rule-based classifier).

Among the multivariate techniques, we considered *ReliefF* (RF) [29] and *SVM-embedded feature selection* [30]. The basic idea of RF is to estimate the relevance of features based on their ability to distinguish between instances that are near to each other. Instead, SVM-embedded feature selection uses a linear SVM classifier to derive a weight for each feature. Then, based on their weights, the features can be ordered from the most important to the less important (SVM_ONE approach). Moreover, a backward elimination strategy can be adopted which iteratively removes the features with the lowest weights and repeats the overall weighting process on the remaining features (SVM_RFE approach). The fraction of features removed at each iteration, 10% in our experiments, greatly influences the computational complexity of the method.

For all the above feature selection techniques we used the implementation provided by the WEKA machine learning environment [31]. To systematically evaluate the degree of overlapping between the gene subsets selected by the considered rankers, we developed a software module that interfaces with WEKA. In what concerns the functional aspects, the similarity analysis was performed by the online tools available at [32]. 

As regards the evaluation of predictive performance and stability of selected gene subsets, the parameters of our methodology were set as follows: a number *P* = 20 of reduced datasets were extracted from the original dataset, and each reduced dataset contains a fraction *f* = 0.9 of the original samples. The ranked lists produced on these datasets by the *M* = 8 rankers were cut at different threshold points (*t* = 5, *t* = 10, *t* = 20, *t* = 30, etc.) as to evaluate stability and predictive performance for gene subsets of increasing size. In evaluating the predictive performance, we used as induction algorithm a linear SVM classifier, which is widely considered the “best of class” method in the context of microarray data analysis; specifically, we employed the SVM implementation provided by WEKA.

## 4. Experimental Results and Discussion

In this section we present and discuss the most significant experimental results. First, we concentrate on findings from the similarity analysis among the gene subsets selected by different rankers (see Section 2.1); then we examine the results of the joint evaluation of stability and predictive performance of selected gene subsets (see Section 2.2).

### 4.1. Results of Similarity Analysis

The similarity analysis was first performed in terms of gene overlapping; that is, we used the I-overlap index to compare, in pairs, the subsets produced by the ranking methods presented in Section 3 (*χ* ^2^, IG, SU, GR, OR, RF, SVM_RFE, and SVM_ONE).  Table 1 shows the results of this comparison for gene subsets of size *t* = 10. Specifically, for each of the considered datasets, that is, (a) *Colon*, (b) *Leukemia*, and (c) *Prostate*, the results are represented in a matricial form: each cell contains the I-overlap value for the pair of subsets selected by the rankers in the corresponding row and column. Different shades of gray are used to highlight different similarity ranges: the darker the gray, the higher the similarity values. The average similarity over all pairwise comparisons is 0.28 for *Colon*, 0.49 for *Leukemia*; and 0.29 for *Prostate* (excluding the cells in the main diagonal where each subset is compared with itself).

Results in Table 1 give useful insight about the pattern of agreement of the considered methods. As regards the univariate approaches (i.e., *χ* ^2^, IG, SU, GR, and OR), first evidence is that the *χ* ^2^ statistic produces results quite similar to entropic methods IG and SU (I-overlap ≥ 0.67 for all the considered benchmarks). The other entropic method, that is, GR, turns out very similar to both IG and SU in the *Leukemia* dataset but exhibits a somewhat different behavior in the other datasets, especially *Colon* which is recognized as a more noisy benchmark. Globally, the univariate methods are more similar to each other than to the multivariate approaches (i.e., RF, SVM_RFE, and SVM_ONE). In particular, the SVM-embedded feature selection produces feature subsets that overlap to a small extent (or do not overlap at all) with the subsets selected by other methods. 

As a further step, the same gene subsets of size *t* = 10 were compared in functional terms based on the molecular function GO annotations of genes in each subset. Results of this comparison are shown in Table 2. Again, for each dataset, results are reported in a matricial form: each cell contains here the I-functional value for a pair of subsets selected by the considered ranking methods. The average similarity is 0.78 for *Colon*, 0.86 for *Leukemia*, and 0.79 for *Prostate*.

Though similarity values in Tables 1 and 2 are not directly comparable, due to the different similarity measures, the ontological analysis shows that the functions captured by different gene subsets can be similar, despite a little degree of overlapping between these subsets. Interestingly, even two subsets with no genes in common may exhibit a fairly high level of functional similarity. Hence, there may be common functions shared across different subsets that are not apparent on the individual gene level. This helps explain why different selection methods can produce different biological signatures: these signatures may be in some way “consistent,” even if they do not contain the same genes.

### 4.2. Results about Stability and Predictive Performance

**Figure 3:**
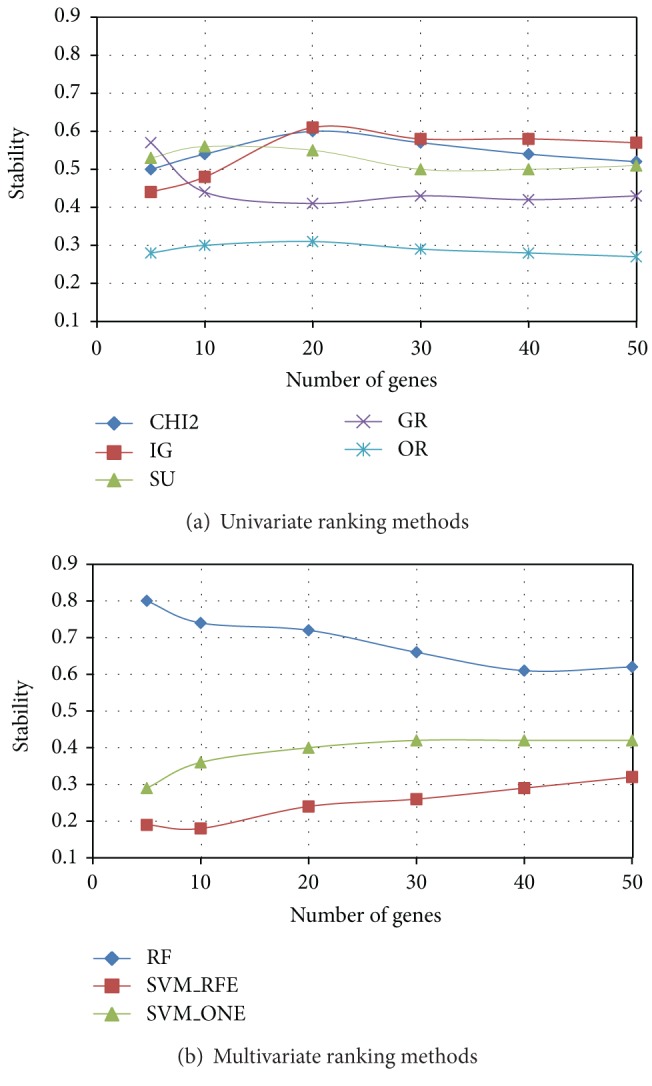
*Colon *dataset: stability versus number of genes.

**Figure 4:**
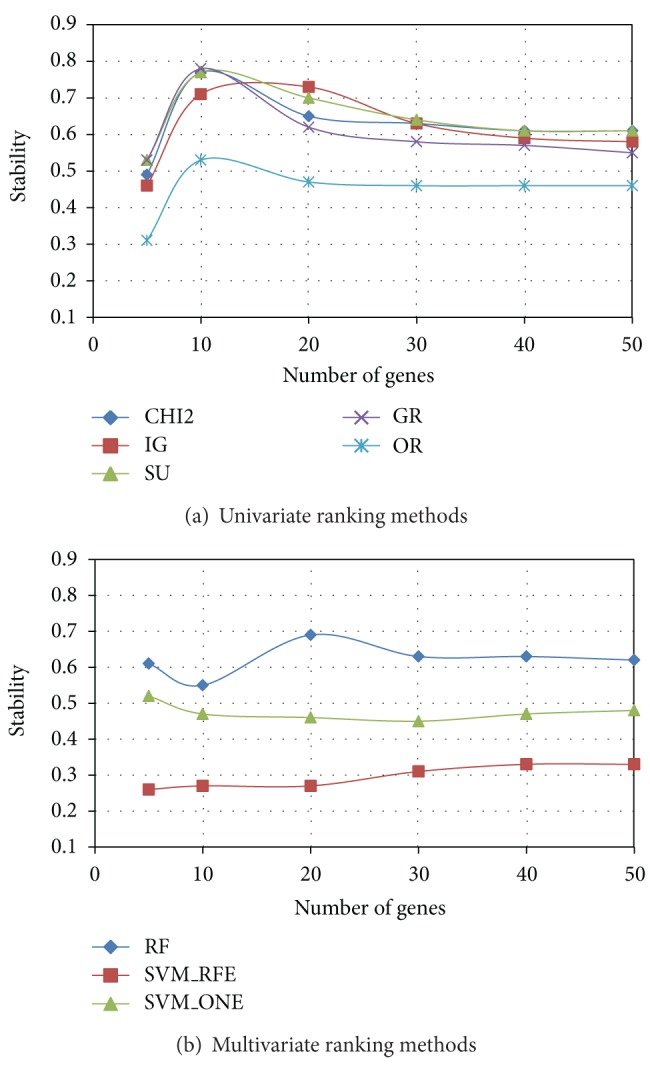
*Leukemia *dataset: stability versus number of genes.

**Figure 5:**
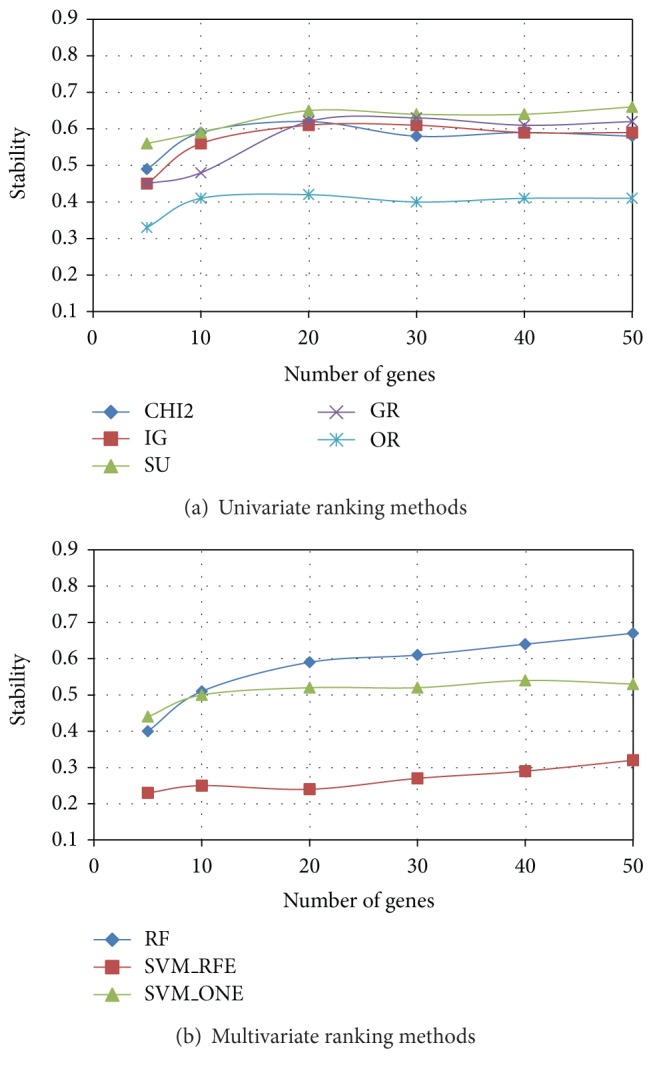
*Prostate *dataset: stability versus number of genes.

After evaluating the degree of similarity/dissimilarity among the outcomes of different ranking methods, we empirically examined the implications of these differences in terms of both stability and predictive performance of selected gene subsets. In Figures 3, 4, and 5 we summarize, respectively, for *Colon*, *Leukemia*, and *Prostate* datasets, the results of stability analysis on gene subsets of increasing size. As explained in Section 2.2, the stability value was obtained, for a given ranking method, as the average similarity (I-overlap) among the gene subsets selected by this method from a number *P* = 20 of reduced datasets randomly drawn from the original dataset. 

Among the univariate approaches (*χ* ^2^, IG, SU, GR, and OR), *χ* ^2^ and the entropic methods IG and SU exhibit, in each dataset, a similar trend in terms of stability, while GR slightly deviates from the other entropic methods in the *Colon* dataset. The worst performing univariate method is OR, which always results in a poor stability irrespective of the number of genes included in the subset. Among the multivariate approaches (RF, SVM_RFE, and SVM_ONE), RF outperforms the SVM-embedded feature selection in each of the benchmarks here considered. In particular, though SVM_RFE is known in the literature [30, 33] as a very effective feature selection technique, it exhibits the worst behavior in terms of stability.

**Figure 6:**
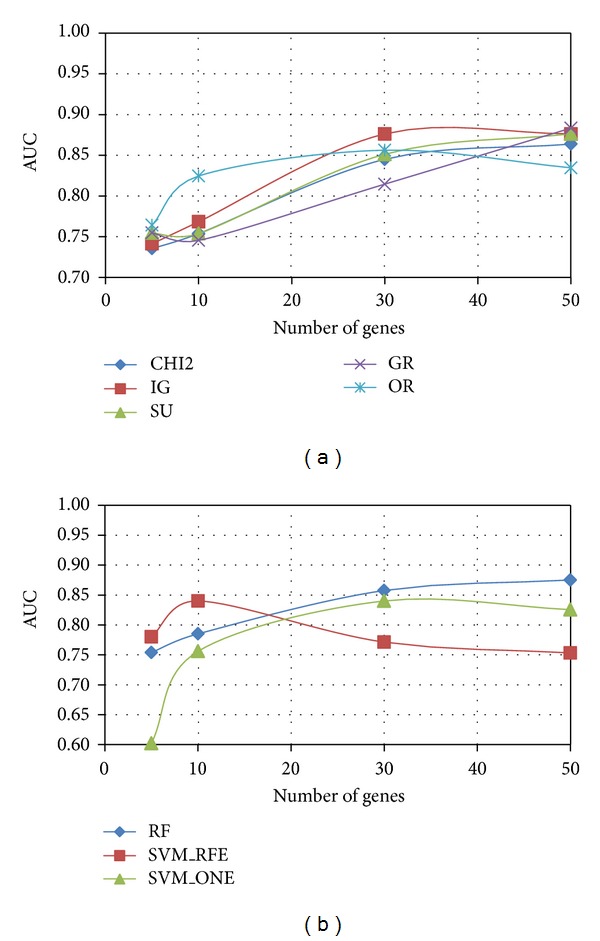
*Colon* dataset: AUC versus number of genes.

**Figure 7:**
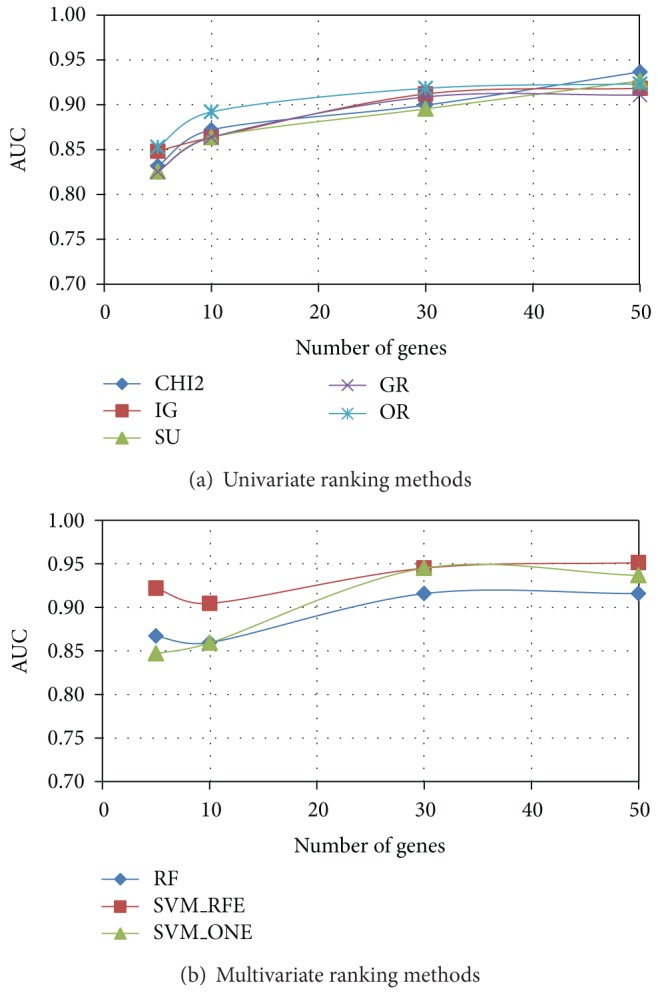
*Leukemia* dataset: AUC versus number of genes.

**Figure 8:**
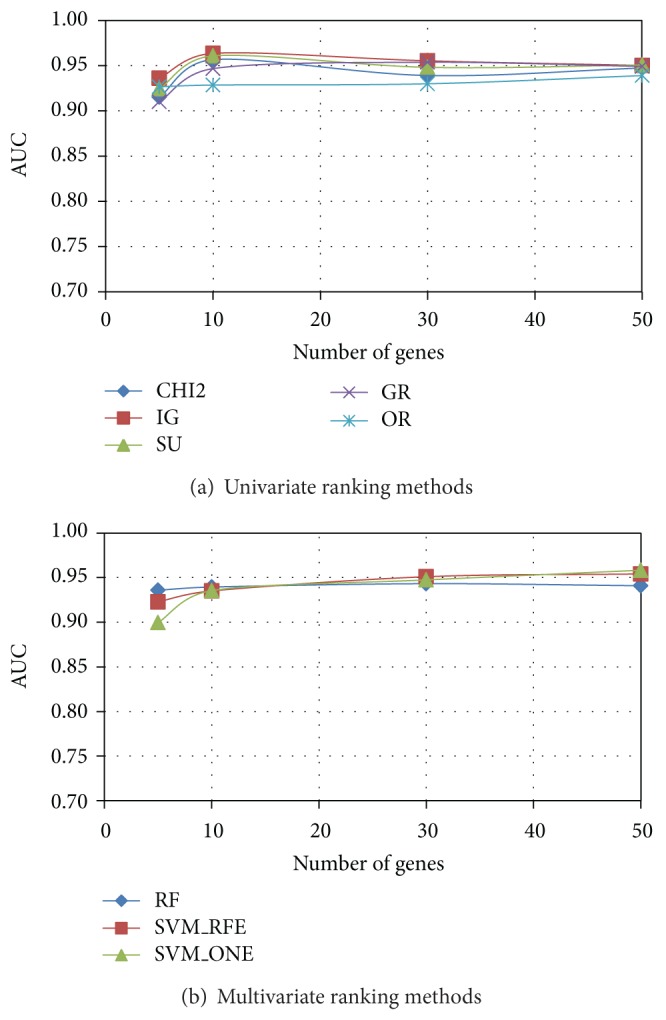
*Prostate* dataset: AUC versus number of genes.

As regards the evaluation of predictive performance, we trained a linear SVM classifier on each of the *P* = 20 gene subsets (of a given size) selected by a given ranking method from the reduced datasets randomly drawn from the original dataset: these reduced datasets serve at this stage as training sets. The average AUC performance, measured on the independent test sets (see Section 2.2), is shown in Figure 6 (*Colon*), Figure 7 (*Leukemia*), and Figure 8 (*Prostate*) for both univariate (*χ* ^2^, IG, SU, GR, and OR) and multivariate methods (RF, SVM_RFE, and SVM_ONE); in each figure, the AUC trend is reported for gene subsets of increasing size.

As we can see, *χ* ^2^ and entropic methods globally exhibit a similar behavior, almost coincident in *Leukemia* and *Prostate* datasets, with a slight superiority of IG in the more problematic *Colon* dataset: here GR turns out to be, for subsets of small-moderate size (<40), the worst performing univariate method. Interestingly, the OR method performs well in terms of AUC (even better than other univariate approaches, for subsets of small size, in both *Colon* and *Leukemia* datasets), though its behavior in terms of stability is quite poor. 

As regards the AUC performance of multivariate approaches, there is no method that univocally outperforms the others, contrary to what is observed in the stability analysis. In the *Prostate* dataset, indeed, the three multivariate methods are almost equivalent, while greater differences can be observed in the *Leukemia* dataset and, even more, in the *Colon* dataset. However, it is worth remarking that SVM_RFE, in all the considered benchmarks, is very effective in identifying small subsets of highly predictive genes, despite its very low stability. We also observe that RF, which is the more stable multivariate method, has globally a good performance also in terms of AUC.

To conclude, a number of observations can be drawn from the joint analysis of stability and AUC patterns of the eight ranking methods considered in this study. As a first point, a high level of agreement exists between the behavior of the statistical approach *χ* ^2^ and the behavior of entropic approaches, especially SU and IG. However, in the *Colon* dataset (which is recognized as a more challenging benchmark), the entropic method GR performs worse, probably due to its higher sensitivity to noise [34]. Moreover, it is interesting to highlight that the less stable methods, that is, OR in the univariate category and SVM_RFE in the multivariate category, are both capable of selecting small-sized subsets of highly predictive genes. Such cases of instability coupled with high predictive performance could be explained in terms of redundancy within the full set of genes: the dataset may contain various markers that are highly correlated which might lead the algorithm to select different genes on different samples [7]. Globally, *χ* ^2^, SU, and IG, representatives of univariate approaches, and RF, representative of multivariate approaches, seem to best satisfy the objective of jointly optimized stability and effectiveness of selected biomarkers.

## 5. Concluding Remarks and Future Research Directions

A methodology has been presented for comparing the outcomes of different feature selection techniques in the context of biomarker discovery. Leveraging on best practices from the literature, the proposed approach enables a multifaceted evaluation of the degree of consistency among the genetic signatures selected by different techniques. 

As a case study, three public benchmarks have been used to empirically evaluate the pattern of agreement of some popular biomarker discovery methods. For future work, further experiments will be performed using more datasets as well as different selection methods. Moreover, different similarity measures could be incorporated in our methodology, especially in what concerns the evaluation of the functional similarity among signatures, which is recognized as a controversial research problem [15].

We also observe that the approach presented in this paper can be a starting point for defining a suitable “ensemble” strategy for biomarker selection. Indeed, recent research efforts attempt to combine multiple feature selection techniques, instead of using a single one, in order to overcome the intrinsic limitations of each technique and obtain a more reliable “consensus” result (e.g., a consensus ranking or a consensus subset containing the most frequently selected features). However, this combination is often made on an “ad hoc” basis [35–39], depending on the specific problem at hand, without considering the degree of diversity/similarity of the involved methods. In our opinion, instead, this important aspect should not be neglected: it would not be beneficial, indeed, to combine two or more techniques that give almost identical results. On the contrary, in an ensemble perspective, the aim should be to reach a consensus result among methods that are capable of giving different and complementary representations of the considered domain. On this premise, our future research will explore suitable ways of combining biomarker selection techniques on the basis of their degree of diversity/similarity, as assessed according to the approach here discussed.

## Figures and Tables

**Table 1a:** (a)  *Colon*  dataset

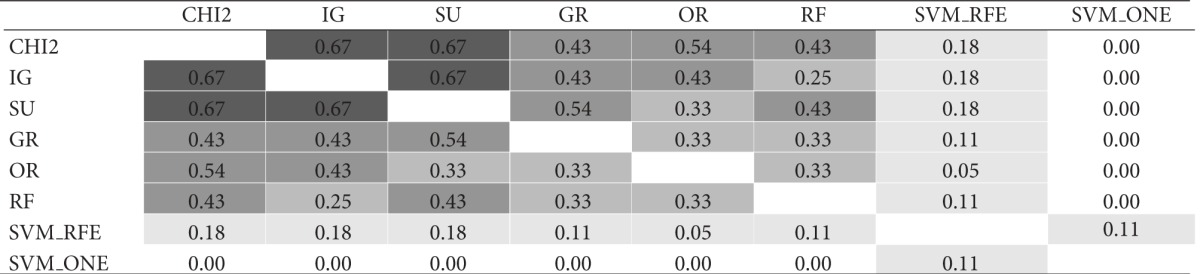

**Table 1b:** (b)  *Leukemia*  dataset

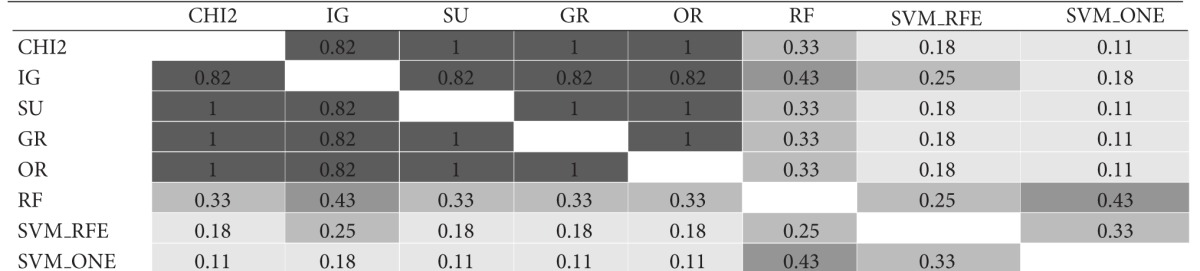

**Table 1c:** (c)  *Prostate*  dataset

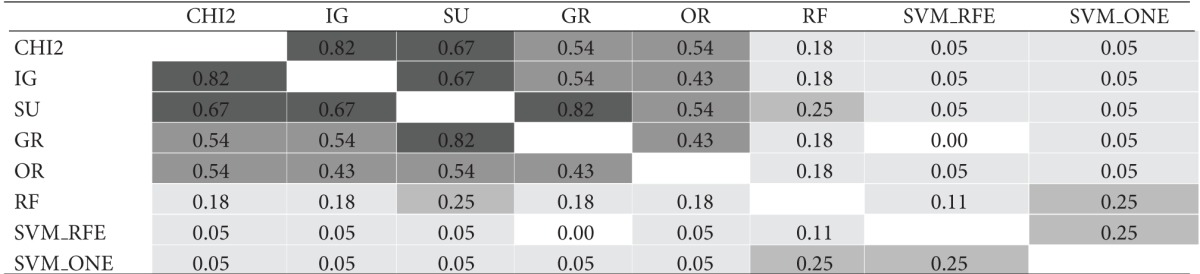

**Table 2a:** (a) *Colon* dataset

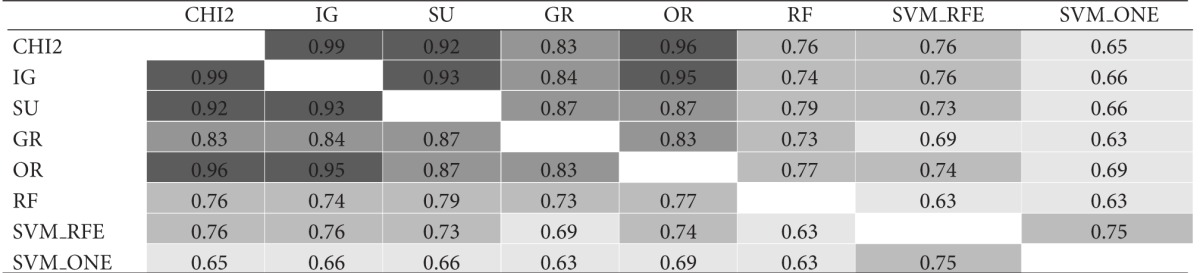

**Table 2b:** (b) *Leukemia* dataset

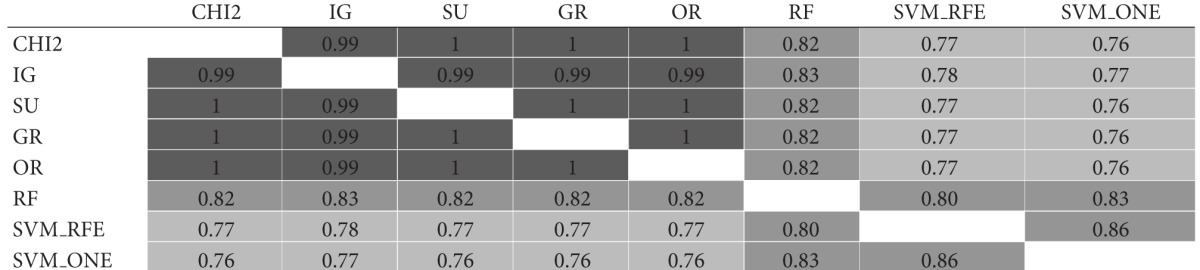

**Table 2c:** (c) *Prostate* dataset